# Aqua­tri­fluorido­boron–1,3-dioxolan-2-one (1/2)

**DOI:** 10.1107/S2414314623000627

**Published:** 2023-01-26

**Authors:** Kristian Radan, Juan Forero-Saboya, Alexandre Ponrouch, Matic Lozinšek

**Affiliations:** a Jožef Stefan Institute, Jamova cesta 39, 1000 Ljubljana, Slovenia; b Institut de Ciència de Materials de Barcelona, ICMAB-CSIC, Campus UAB 08193, Bellaterra, Spain; Vienna University of Technology, Austria

**Keywords:** aqua­tri­fluorido­boron, ethyl­ene carbonate, co-crystal, crystal structure

## Abstract

The crystal structure of the co-crystal of aqua­tri­fluorido­boron with two ethyl­ene carbonate (systematic name: 1,3-dioxolan-2-one) mol­ecules, BF_3_H_2_O·2OC(OCH_2_)_2_, was determined by low-temperature single-crystal X-ray diffraction. The co-crystal crystallizes in the ortho­rhom­bic space group *P*2_1_2_1_2_1_ with four formula units per unit cell. The asymmetric unit consists of an aqua­tri­fluorido­boron mol­ecule and two ethyl­ene carbonate mol­ecules, connected by O—H⋯O=C hydrogen bonds. This crystal structure is an inter­esting example of a superacidic BF_3_H_2_O species co-crystallized with an organic carbonate.

## Abstract 2

Aqua­tri­fluorido­boron and ethyl­ene carbonate form a 1:2 co-crystal with a C=O⋯H—O—H⋯O=C hydrogen-bonding motif.

## Graphical abstract

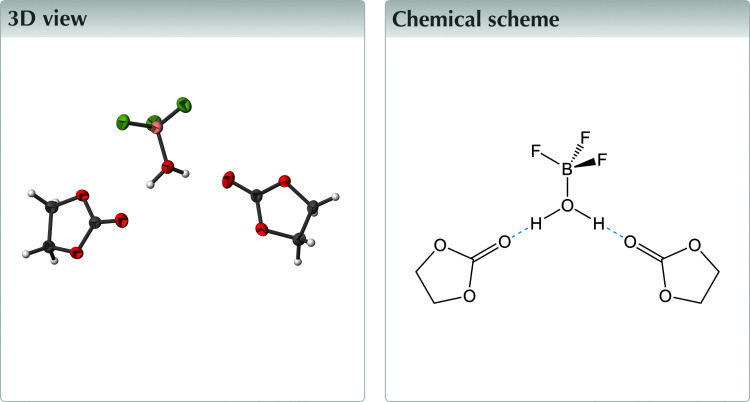

## Structure description

Adducts synthesized from boron trifluoride and various organic carbonates have been reported as potential functional electrolyte additives for secondary (rechargeable) lithium-ion batteries (Eisele *et al.*, 2020 ▸), and have been shown to modify the electrode surfaces, resulting in reduced cell resistance and better capacity retention at high current rates. Recently, the use of BF_3_-based additives has been extended to divalent-metal batteries, namely calcium-ion batteries (Forero-Saboya *et al.*, 2021 ▸; Bodin *et al.*, 2023 ▸), where their decomposition into boron-crosslinked polymeric matrices in the passivation layer was found to be crucial for calcium plating and stripping. Such BF_3_ adducts are moisture sensitive and readily hydrolyze to form BF_3_H_2_O (Simonov *et al.*, 1996 ▸; Fonari *et al.*, 1997 ▸). The title co-crystal formed from the boron trifluoride–ethyl­ene carbonate (1/1) adduct, BF_3_·OC(OCH_2_)_2_, upon exposure to moisture.

**Figure 1:**
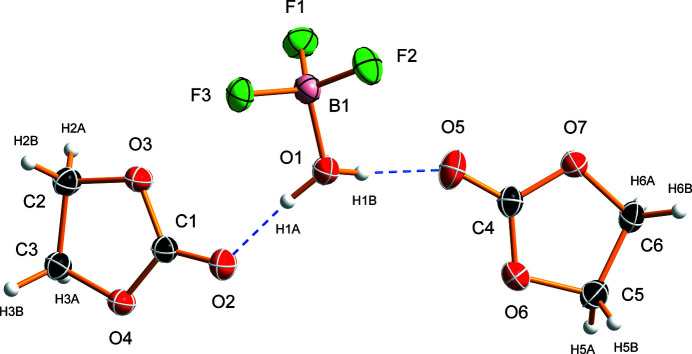
The asymmetric unit and the atom-labelling scheme of the BF_3_H_2_O·2OC(OCH_2_)_2_ co-crystal. Anisotropic displacement ellipsoids are drawn at the 50% probability level, hydrogen atoms are depicted as spheres of arbitrary radius, and hydrogen bonds are indicated by blue dashed lines.

The BF_3_H_2_O·2OC(OCH_2_)_2_ co-crystal crystallizes in the ortho­rhom­bic Sohncke space group *P*2_1_2_1_2_1_ with one aqua­tri­fluorido­boron and two ethyl­ene carbonate mol­ecules in the asymmetric unit (Fig. 1 ▸). The two OC(OCH_2_)_2_ mol­ecules have an essentially identical mol­ecular shape (slightly twisted), which also agrees well with the crystal structure determination of 1,3-dioxolan-2-one (Atterberry & Bond, 2019 ▸). The B—O and B—F bond lengths [1.5236 (18) Å and 1.3718 (18)–1.3760 (17) Å, respectively] in the BF_3_H_2_O mol­ecule of the title co-crystal are similar to those found in BF_3_H_2_O (Mootz & Steffen, 1981*a* ▸), BF_3_H_2_O·H_2_O (Mootz & Steffen, 1981*b* ▸), BF_3_H_2_O·C_4_H_8_O_2_ (Barthen & Frank, 2019 ▸), or adducts of BF_3_ and organic carbonates (Bodin *et al.*, 2023 ▸). The F—B—F angles [110.75 (12)–112.57 (12)°] are larger than the O—B—F angles, with the angle involving F1 [109.23 (11)°] being significantly larger than the other two angles [105.47 (11)° and 106.41 (12)°]. The hydrogen atoms of the H_2_O moiety in the BF_3_H_2_O adduct are inclined toward the F1 atom, with the angle between the B—O bond and the plane defined by the water atoms being 128 (2)°. The overall shape of the BF_3_ moiety in BF_3_H_2_O in terms of bond lengths and angles is similar to that of the BF_4_^−^ anion (Lozinšek, 2021 ▸).

Aqua­tri­fluorido­boron is stabilized in the solid state by hydrogen-bonding inter­actions with oxygen hydrogen-bond acceptors, such as 1,4-dioxane (Barthen & Frank, 2019 ▸) or crown ethers (Bott *et al.*, 1991 ▸; Simonov *et al.*, 1996 ▸; Fonari *et al.*, 1997 ▸; Gelmboldt *et al.*, 2012 ▸). In the present case, the BF_3_H_2_O mol­ecule is hydrogen-bonded to the carbonyl oxygen atoms of the two ethyl­ene carbonate mol­ecules, forming a C=O⋯H—O—H⋯O=C fragment with a *D* ^2^_2_(5) graph-set motif (Etter, 1990 ▸) and O⋯O distances of 2.5637 (15) Å and 2.5985 (15) Å (Table 1 ▸, Figs. 1 ▸ and 2 ▸). A similar hydrogen-bonding motif was observed in the crystal structure of the BF_3_H_2_O·2Ph_3_PO co-crystal (Chekhlov, 2005 ▸).

## Synthesis and crystallization

Single crystals of the BF_3_H_2_O·2OC(OCH_2_)_2_ co-crystal were discovered when a crystalline sample of the air-sensitive BF_3_·OC(OCH_2_)_2_ adduct was examined under a protective cold nitro­gen stream at about −50 °C. The BF_3_·OC(OCH_2_)_2_ compound was synthesized from dry ethyl­ene carbonate and BF_3_ gas under anhydrous conditions, as described previously (Bodin *et al.*, 2023 ▸). Platelet-shaped co-crystals of BF_3_H_2_O·2OC(OCH_2_)_2_ were located in a droplet at the tip of the aluminium trough (Veith & Bärnighausen, 1974 ▸) of the low-temperature crystal mounting apparatus, which likely formed by an inadvertent introduction of a small amount of moisture. Selected crystals were mounted on the diffractometer employing a previously described procedure for mounting crystals at low temperatures (Lozinšek *et al.*, 2021 ▸). The crystals melted and turned into droplets when exposed to air at room temperature.

## Refinement

Crystal data, data collection, and structure refinement details are summarized in Table 2 ▸. Positions and isotropic thermal displacement parameters of hydrogen atoms were freely refined (Cooper *et al.*, 2010 ▸).

## Supplementary Material

Crystal structure: contains datablock(s) I. DOI: 10.1107/S2414314623000627/wm4182sup1.cif

Structure factors: contains datablock(s) I. DOI: 10.1107/S2414314623000627/wm4182Isup2.hkl

CCDC reference: 2237804

Additional supporting information:  crystallographic information; 3D view; checkCIF report

## Figures and Tables

**Figure 2:**
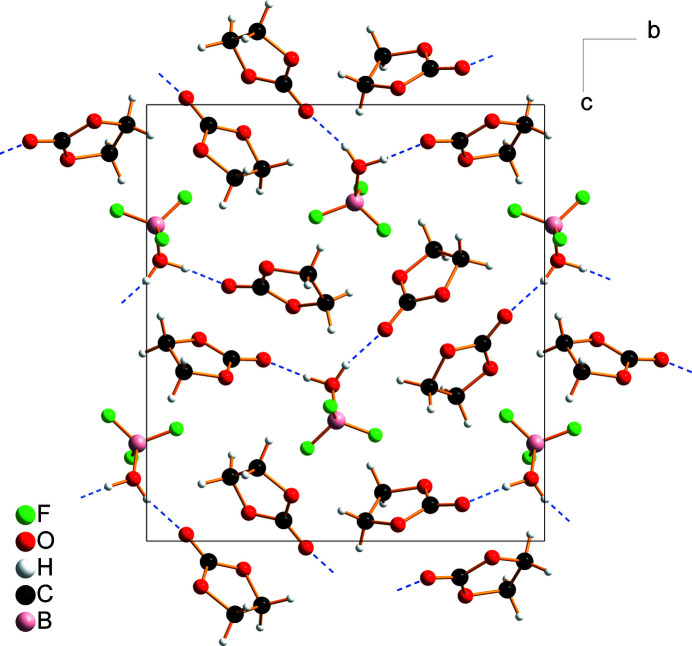
Crystal packing of BF_3_H_2_O·2OC(OCH_2_)_2_ viewed along [100]. Hydrogen bonds are indicated by blue dashed lines.

**Table 1:** Hydrogen-bond geometry (Å, °)

*D*—H⋯*A*	*D*—H	H⋯*A*	*D*⋯*A*	*D*—H⋯*A*
O1—H1*A*⋯O2	0.90 (3)	1.67 (3)	2.5637 (15)	175 (3)
O1—H1*B*⋯O5	0.82 (3)	1.79 (3)	2.5985 (15)	166 (2)

**Table 2:** Experimental details

Crystal data	
Chemical formula	2C_3_H_4_O_3_·H_2_BF_3_O
*M*_r_	261.95
Crystal system, space group	Orthorhombic, *P*2_1_2_1_2_1_
Temperature (K)	150
*a*, *b*, *c* (Å)	5.44197 (4), 13.09134 (8), 14.36102 (9)
*V* (Å^3^)	1023.12 (1)
*Z*	4
Radiation type	Cu *K*α
μ (mm^−1^)	1.65
Crystal size (mm)	0.18 × 0.08 × 0.05

Data collection	
Diffractometer	XtaLAB Synergy, Dualflex, Eiger2 R CdTe 1M
Absorption correction	Gaussian (*CrysAlis PRO*; Rigaku OD, 2022 ▸)
*T*_min_, *T*_max_	0.663, 1.000
No. of measured, independent and observed [*I* > 2σ(*I*)] reflections	34542, 2134, 2100
*R*_int_	0.038
(sin θ/λ)_max_ (Å^−1^)	0.630

Refinement	
*R*[*F* ^2^ > 2σ(*F* ^2^)], *wR*(*F* ^2^), *S*	0.019, 0.050, 1.04
No. of reflections	2134
No. of parameters	195
H-atom treatment	All H-atom parameters refined
Δρ_max_, Δρ_min_ (e Å^−3^)	0.11, −0.13
Absolute structure	Flack *x* determined using 853 quotients [(*I* ^+^)−(*I* ^−^)]/[(*I* ^+^)+(*I* ^−^)] (Parsons *et al.*, 2013 ▸)
Absolute structure parameter	−0.05 (3)

Computer programs: *CrysAlis PRO* (Rigaku OD, 2022 ▸), *SHELXT* (Sheldrick, 2015*a* ▸), *SHELXL* (Sheldrick, 2015*b* ▸), *OLEX2* (Dolomanov *et al.*, 2009 ▸), *DIAMOND* (Brandenburg, 2005 ▸) and *publCIF* (Westrip, 2010 ▸).

## full crystallographic data

### Crystal data

**Table:**

2C_3_H_4_O_3_·H_2_BF_3_O	*D*_x_ = 1.701 Mg m^−^^3^
*M**_r_* = 261.95	Cu *K*α radiation, λ = 1.54184 Å
Orthorhombic, *P*2_1_2_1_2_1_	Cell parameters from 24984 reflections
*a* = 5.44197 (4) Å	θ = 4.6–75.5°
*b* = 13.09134 (8) Å	µ = 1.65 mm^−^^1^
*c* = 14.36102 (9) Å	*T* = 150 K
*V* = 1023.12 (1) Å^3^	Plate, clear colourless
*Z* = 4	0.18 × 0.08 × 0.05 mm
*F*(000) = 536	

### Data collection

**Table:**

XtaLAB Synergy, Dualflex, Eiger2 R CdTe 1M diffractometer	2134 independent reflections
Radiation source: micro-focus sealed X-ray tube, PhotonJet (Cu) X-ray Source	2100 reflections with *I* > 2σ(*I*)
Mirror monochromator	*R*_int_ = 0.038
Detector resolution: 13.3333 pixels mm^-1^	θ_max_ = 76.1°, θ_min_ = 4.6°
ω scans	*h* = −6→6
Absorption correction: gaussian (*CrysAlisPro*; Rigaku OD, 2022)	*k* = −15→16
*T*_min_ = 0.663, *T*_max_ = 1.000	*l* = −18→18
34542 measured reflections	

### Refinement

**Table:**

Refinement on *F*^2^	All H-atom parameters refined
Least-squares matrix: full	*w* = 1/[σ^2^(*F*_o_^2^) + (0.0311*P*)^2^ + 0.1146*P*] where *P* = (*F*_o_^2^ + 2*F*_c_^2^)/3
*R*[*F*^2^ > 2σ(*F*^2^)] = 0.019	(Δ/σ)_max_ < 0.001
*wR*(*F*^2^) = 0.050	Δρ_max_ = 0.11 e Å^−^^3^
*S* = 1.04	Δρ_min_ = −0.13 e Å^−^^3^
2134 reflections	Extinction correction: SHELXL (Sheldrick, 2015b), F_c_^*^ = kF_c_[1+0.001xF_c_^2^λ^3^/sin(2θ)]^–1/4^
195 parameters	Extinction coefficient: 0.0035 (5)
0 restraints	Absolute structure: Flack *x* determined using 853 quotients [(*I*^+^)–(*I*^–^)]/[(*I*^+^)+(*I*^–^)] (Parsons *et al.*, 2013)
Primary atom site location: dual	Absolute structure parameter: −0.05 (3)
Hydrogen site location: difference Fourier map	

### Special details

**Table:**

Geometry. All esds (except the esd in the dihedral angle between two l.s. planes) are estimated using the full covariance matrix. The cell esds are taken into account individually in the estimation of esds in distances, angles and torsion angles; correlations between esds in cell parameters are only used when they are defined by crystal symmetry. An approximate (isotropic) treatment of cell esds is used for estimating esds involving l.s. planes.

### Fractional atomic coordinates and isotropic or equivalent isotropic displacement parameters (Å^2^)

**Table:**

	*x*	*y*	*z*	*U*_iso_*/*U*_eq_	
F1	0.05387 (16)	0.46158 (7)	0.69151 (6)	0.0345 (2)	
F2	0.31541 (19)	0.57475 (6)	0.75716 (6)	0.0347 (2)	
F3	0.35784 (17)	0.40399 (6)	0.78723 (6)	0.0320 (2)	
O1	0.4683 (2)	0.46651 (8)	0.64281 (7)	0.0283 (2)	
H1A	0.518 (5)	0.404 (2)	0.6252 (19)	0.071 (8)*	
H1B	0.432 (5)	0.5005 (19)	0.5968 (17)	0.052 (6)*	
O2	0.6234 (2)	0.29312 (8)	0.58425 (7)	0.0312 (2)	
O3	0.29867 (18)	0.19871 (7)	0.62526 (7)	0.0266 (2)	
O4	0.59130 (18)	0.13169 (7)	0.53775 (7)	0.0255 (2)	
C1	0.5104 (2)	0.21312 (10)	0.58256 (9)	0.0232 (3)	
C2	0.2189 (3)	0.09379 (11)	0.61128 (10)	0.0267 (3)	
H2A	0.062 (4)	0.0949 (14)	0.5867 (13)	0.033 (5)*	
H2B	0.220 (3)	0.0629 (13)	0.6729 (12)	0.025 (4)*	
C3	0.4125 (3)	0.04997 (10)	0.54606 (10)	0.0249 (3)	
H3A	0.349 (4)	0.0354 (14)	0.4844 (13)	0.031 (4)*	
H3B	0.497 (4)	−0.0097 (15)	0.5697 (12)	0.030 (5)*	
B1	0.2884 (3)	0.47702 (12)	0.72354 (11)	0.0252 (3)	
O5	0.3601 (2)	0.59963 (8)	0.51501 (8)	0.0359 (3)	
O6	0.60783 (19)	0.63618 (7)	0.39559 (7)	0.0280 (2)	
O7	0.31353 (19)	0.74337 (7)	0.43534 (6)	0.0266 (2)	
C4	0.4246 (3)	0.65572 (10)	0.45246 (9)	0.0240 (3)	
C5	0.6409 (3)	0.72193 (11)	0.33235 (10)	0.0305 (3)	
H5B	0.793 (4)	0.7521 (16)	0.3486 (14)	0.040 (5)*	
H5A	0.649 (4)	0.6923 (14)	0.2694 (14)	0.038 (5)*	
C6	0.4207 (3)	0.78959 (11)	0.35281 (10)	0.0291 (3)	
H6B	0.470 (3)	0.8616 (15)	0.3687 (13)	0.032 (4)*	
H6A	0.296 (4)	0.7853 (15)	0.3028 (14)	0.040 (5)*	

### Atomic displacement parameters (Å^2^)

**Table:**

	*U*^11^	*U*^22^	*U*^33^	*U*^12^	*U*^13^	*U*^23^
F1	0.0260 (4)	0.0419 (5)	0.0356 (4)	−0.0012 (4)	−0.0027 (3)	0.0046 (4)
F2	0.0475 (5)	0.0265 (4)	0.0300 (4)	0.0037 (4)	−0.0046 (4)	−0.0043 (3)
F3	0.0379 (5)	0.0309 (4)	0.0273 (4)	0.0026 (4)	−0.0013 (4)	0.0085 (3)
O1	0.0324 (5)	0.0254 (5)	0.0271 (5)	0.0034 (4)	0.0049 (4)	0.0046 (4)
O2	0.0325 (5)	0.0237 (4)	0.0373 (5)	−0.0033 (4)	0.0051 (5)	−0.0004 (4)
O3	0.0256 (5)	0.0243 (4)	0.0299 (5)	0.0006 (4)	0.0066 (4)	−0.0026 (4)
O4	0.0236 (5)	0.0243 (4)	0.0285 (5)	0.0008 (4)	0.0043 (4)	−0.0025 (4)
C1	0.0240 (6)	0.0236 (6)	0.0220 (6)	0.0025 (5)	0.0007 (5)	0.0016 (5)
C2	0.0243 (7)	0.0258 (6)	0.0300 (7)	−0.0032 (5)	0.0023 (6)	−0.0027 (5)
C3	0.0236 (6)	0.0234 (6)	0.0275 (6)	−0.0009 (5)	−0.0003 (5)	−0.0010 (5)
B1	0.0282 (8)	0.0253 (7)	0.0220 (7)	0.0020 (6)	−0.0007 (6)	0.0015 (5)
O5	0.0459 (6)	0.0315 (5)	0.0303 (5)	−0.0073 (5)	−0.0016 (5)	0.0098 (4)
O6	0.0299 (5)	0.0225 (4)	0.0314 (5)	0.0031 (4)	0.0017 (4)	−0.0006 (4)
O7	0.0303 (5)	0.0240 (4)	0.0255 (4)	0.0035 (4)	0.0055 (4)	0.0024 (4)
C4	0.0278 (7)	0.0212 (6)	0.0229 (6)	−0.0023 (5)	−0.0024 (5)	0.0002 (5)
C5	0.0343 (8)	0.0271 (7)	0.0302 (7)	−0.0041 (6)	0.0083 (6)	0.0000 (6)
C6	0.0355 (8)	0.0244 (7)	0.0273 (6)	0.0000 (6)	0.0034 (6)	0.0069 (5)

### Geometric parameters (Å, º)

**Table:**

F1—B1	1.3718 (18)	C2—C3	1.522 (2)
F2—B1	1.3753 (17)	C3—H3A	0.969 (19)
F3—B1	1.3760 (17)	C3—H3B	0.97 (2)
O1—H1A	0.90 (3)	O5—C4	1.2122 (17)
O1—H1B	0.82 (3)	O6—C4	1.3139 (18)
O1—B1	1.5236 (18)	O6—C5	1.4550 (17)
O2—C1	1.2147 (17)	O7—C4	1.3200 (16)
O3—C1	1.3187 (16)	O7—C6	1.4530 (17)
O3—C2	1.4545 (16)	C5—H5B	0.95 (2)
O4—C1	1.3208 (16)	C5—H5A	0.98 (2)
O4—C3	1.4512 (17)	C5—C6	1.519 (2)
C2—H2A	0.92 (2)	C6—H6B	1.01 (2)
C2—H2B	0.972 (18)	C6—H6A	0.99 (2)
			
H1A—O1—H1B	110 (2)	F1—B1—O1	109.23 (11)
B1—O1—H1A	119.4 (18)	F2—B1—F3	112.57 (12)
B1—O1—H1B	114.2 (17)	F2—B1—O1	106.41 (12)
C1—O3—C2	109.38 (10)	F3—B1—O1	105.47 (11)
C1—O4—C3	109.35 (10)	C4—O6—C5	109.37 (11)
O2—C1—O3	123.81 (12)	C4—O7—C6	109.27 (11)
O2—C1—O4	122.46 (12)	O5—C4—O6	124.23 (13)
O3—C1—O4	113.73 (12)	O5—C4—O7	122.13 (14)
O3—C2—H2A	108.3 (12)	O6—C4—O7	113.64 (11)
O3—C2—H2B	105.4 (10)	O6—C5—H5B	106.1 (13)
O3—C2—C3	103.55 (11)	O6—C5—H5A	105.9 (11)
H2A—C2—H2B	110.9 (16)	O6—C5—C6	103.38 (11)
C3—C2—H2A	114.2 (12)	H5B—C5—H5A	110.5 (17)
C3—C2—H2B	113.6 (11)	C6—C5—H5B	113.6 (13)
O4—C3—C2	103.71 (11)	C6—C5—H5A	116.3 (12)
O4—C3—H3A	108.0 (11)	O7—C6—C5	103.40 (11)
O4—C3—H3B	107.7 (11)	O7—C6—H6B	108.2 (11)
C2—C3—H3A	112.9 (12)	O7—C6—H6A	107.1 (12)
C2—C3—H3B	114.7 (11)	C5—C6—H6B	112.2 (11)
H3A—C3—H3B	109.3 (16)	C5—C6—H6A	111.6 (11)
F1—B1—F2	110.75 (12)	H6B—C6—H6A	113.6 (15)
F1—B1—F3	112.08 (13)		
			
O3—C2—C3—O4	5.22 (14)	O6—C5—C6—O7	9.38 (14)
C1—O3—C2—C3	−4.14 (14)	C4—O6—C5—C6	−7.83 (15)
C1—O4—C3—C2	−4.81 (14)	C4—O7—C6—C5	−8.29 (15)
C2—O3—C1—O2	−178.23 (13)	C5—O6—C4—O5	−177.25 (14)
C2—O3—C1—O4	1.26 (15)	C5—O6—C4—O7	2.92 (16)
C3—O4—C1—O2	−178.06 (13)	C6—O7—C4—O5	−176.09 (13)
C3—O4—C1—O3	2.44 (15)	C6—O7—C4—O6	3.74 (16)

### Hydrogen-bond geometry (Å, º)

**Table:**

*D*—H···*A*	*D*—H	H···*A*	*D*···*A*	*D*—H···*A*
O1—H1*A*···O2	0.90 (3)	1.67 (3)	2.5637 (15)	175 (3)
O1—H1*B*···O5	0.82 (3)	1.79 (3)	2.5985 (15)	166 (2)
C3—H3*B*···F3^i^	0.97 (2)	2.474 (19)	3.3085 (16)	144.2 (14)
C6—H6*B*···F1^ii^	1.01 (2)	2.51 (2)	3.3974 (17)	146.4 (14)

Symmetry codes: (i) −*x*+1, *y*−1/2, −*z*+3/2; (ii) *x*+1/2, −*y*+3/2, −*z*+1.
